# Forest Age and Soil Depth Mediate the Effects of Soil and Root Traits on Soil Microbial Community in Plantations

**DOI:** 10.1002/ece3.72264

**Published:** 2025-10-14

**Authors:** Yaxuan Chen, Qianyuan Liu, Yanmei Chen, Changqi Ai, Peipei Jiang

**Affiliations:** ^1^ Hebei Key Laboratory of Environmental Change and Ecological Construction, School of Geographical Sciences Hebei Normal University Shijiazhuang Hebei China; ^2^ Key Lab of Plant Stress Research, College of Life Sciences Shandong Normal University Ji'nan Shandong China

**Keywords:** alpha diversity, forest age, microbial community, root traits, soil depth

## Abstract

The soil microbial community composition and Alpha diversity serve as key indicators of soil quality changes driven by forest development. We explored the variations in soil properties, root traits, microbial communities, and their interrelationships across forest age and soil depth in *Populus tomentosa* , *Platycladus orientalis* , and *Styphnolobium japonicum* plantations. The results showed that the Chao, Shannon, and Pielou_e indices of the soil microbial community increased with forest age. Soil properties and root traits had a stronger influence on the composition of soil bacteria (41.4%) compared to fungi (28.8%). In comparison to root traits (7%–10%), soil properties had a more significant influence (23%–26%) on microbial composition. Soil clay, water content, and conductivity showed positive effects on bacterial diversity and composition, while fungi were mainly affected by soil total phosphorus and soil pH. The influence of root traits on bacterial diversity declined with forest age, whereas the effect of soil properties increased. Fungal diversity was jointly shaped by soil properties and root traits in 13–19a plantations, but mainly by soil properties in 9–12a and 16–36a plantations. With increasing soil depth, the impact of roots on bacterial diversity grew while on fungal diversity diminished. The results highlight the need to account for forest age and soil depth when revealing the association among soil microbial diversity, environmental variation, and root traits.

## Graphical abstract

We determined the soil properties, root traits, and microbial community in two soil depths in *Populus tomentosa* , *Platycladus orientalis* , and *Styphnolobium japonicum* plantations with different forest ages and found that: (1) The influence of soil properties and root traits on the composition of soil bacteria was greater than that of fungi. (2) Compared with root traits, soil properties exhibited greater effects on the composition of bacterial and fungal communities. (3) The influence of root traits on bacterial diversity decreased while that of soil properties increased with increasing forest age. (4) As soil depth increased, the impact of roots on bacterial diversity increased while the impact on fungal diversity decreased.

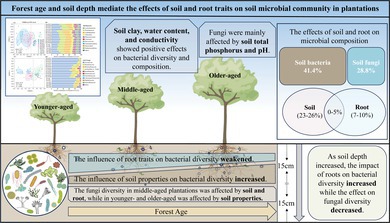

## 1 Introduction

Soil microbes are important participants in the material cycling process, significantly contributing to improving plant productivity, maintaining soil fertility, and ecosystem function (Falkowski et al. 2008). Microbial communities exhibit extremely high sensitivity to environmental changes (Yang and Zhang 2014), whereas changes in their composition and diversity have significant impacts on soil multifunctionality. Forest age and soil depth are two key spatiotemporal gradients that regulate plant–microbe–soil interactions through their effects on soil properties and root traits. Revealing the determinants of soil microbial diversity from the perspectives of age–related changes and soil depths is vital to our understanding of microbial function.

As the forest ages, the physical and chemical properties of the soil change due to litter input, root turnover, and long–term plant–soil feedback. For example, studies have shown that as forest age increases, litter accumulates, thereby increasing soil nutrient content, improving soil texture, and promoting an increase in soil moisture content by enhancing water retention (Chen et al. 2025). Similarly, soil properties also vary with soil depth. However, some studies have found that soil nutrient content decreases with increasing forest age (Peng et al. 2018; Amir et al. 2019). Root systems undergo adaptive changes to suit soil conditions, and root traits such as biomass, specific root length, and root tissue density also adjust according to the needs of trees at different stages of development. Zheng et al. (2023) found that the specific root length and specific surface area of *Cunninghamia lanceolata* decreased with increasing forest age, while Li (2022) found that the specific root length of *Pinus sylvestris* and *Picea koraiensis* increased with increasing forest age. Therefore, the trends in changes in soil properties and root functional traits at different forest ages are inconsistent due to species differences and the complexity of ecological processes. These age–related changes in soil conditions and underground root traits provide an important background for understanding changes in soil microorganisms in forest ecosystems.

The soil microbial communities are influenced by multiple factors, including vegetation type (Hackl et al. 2005), forest age (Cao et al. 2021), and soil physicochemical properties (Zheng et al. 2019). Among different forest types, soil properties exhibited varying patterns of change with increasing forest age, and the corresponding shifts in soil microbial community composition and diversity are often inconsistent, with nutrient content emerging as the primary influencing factor. For instance, Wu et al. (2021) found that in *Populus alba* plantations, the diversity index of soil fungal communities showed a trend of first increasing and then decreasing with forest age (4a, 9a, 15a, 25a, and 30a), with available phosphorus and nitrate nitrogen being significant influencing factors. Similarly, Yuan et al. (2022) reported that soil microbial diversity in middle‐aged (12a) *Cunninghamia lanceolata* plantations was significantly higher than in both younger (6a) and older–aged (25a) plantations, showing a positive correlation with soil organic carbon content. Additional soil properties, including pH (Yu et al. 2021), bulk density (Chatterjee et al. 2009), and moisture content (Zhang et al. 2023), have also been demonstrated to affect soil microorganisms. Furthermore, studies consistently demonstrated that microbial diversity and activity were greater in surface soils compared to deeper layers (Chen et al. 2019; Jiao et al. 2022), likely due to higher nutrient availability and greater fine root density in topsoil. These findings collectively highlight the dynamic responses of soil microbial communities to variations in soil properties across forest types and ages.

Roots drive the cycling of soil carbon and nitrogen by affecting soil microbial communities (Bardgett et al. 2014), which can more accurately predict rhizosphere microbial community composition than leaf traits (Barberán et al. 2015; Cantarel et al. 2015; Sweeney et al. 2021), likely due to their direct interactions with soil and microbes. Previous studies showed that fast‐growing tree species with higher specific root length (SRL) and smaller root diameter (RD) exhibited higher root exudation rates and tended to release more organic carbon (Matthus et al. 2025), thereby promoting an increase in bacterial activity (Meier et al. 2020). Wan et al. (2022) found that higher root nitrogen content (RNC) was associated with a lower ratio of soil fungi to bacteria through measuring 28 subtropical tree species. Spitzer et al. (2021) observed that the relative abundance of arbuscular mycorrhizal fungi exhibited a positive relationship with root carbon content (RCC) and RD, while root branching density and SRL were opposite. The above investigations indicated a strong relationship between soil microorganisms and root traits. However, there is limited research on how the influence of root traits on microbial communities and diversity varies with forest age and soil depth, which limits our understanding of plant‐microbe‐soil interactions and ecosystem functions.

The Taihang Mountain serves as the crucial water conservation area for the North China Plain and plays a significant role in the national strategy for the coordinated development of the Beijing–Tianjin–Hebei region. This study focused on the three forest ages of *Populus tomentosa* , *Platycladus orientalis* , and *Styphnolobium japonicum* plantations in the Taihang Mountain. We examined the interrelationships between soil properties, root traits, and soil microbial communities. Based on previous research, we hypothesize that: (1) the soil nutrient content increases with increasing forest age and decreases with soil depth, while specific root length and specific root area both decrease; (2) with increasing forest age, the Alpha diversity of microbial communities increases, but decreases with the deepening of soil depth; (3) the influence of root traits on soil microbial communities is equivalent to soil properties, and its effect weakens with increasing forest age and soil depth, while the effect of soil properties is anticipated to show the opposite trend.

## 2 Materials and Methods

### 2.1 Experimental Design and Sampling

The study site is located in Lincheng County (114°02′–114°38′ E, 37°20′–37°36′ N), Xingtai City, Hebei Province, ranges in altitude from 38 to 1474 m, averaging 313 m. The area experiences a warm temperate and sub–humid continental monsoon climate. The annual average precipitation is 593.4 mm, and the annual average temperature at the study site from 2020 to 2024 is 13°C. The forest resources in the study region included *Platycladus orientalis* , *Styphnolobium japonicum* , *Populus tomentosa* , *Ulmus pumila* , *Betula platyphylla* , and *Morus alba* . Shrubs are primarily represented by *Vitex negundo* and *Ziziphus jujuba* .

On the basis of field investigation, *P. tomentosa* , *P. orientalis* , and *S. japonicum* plantations were selected as research subjects. The plantations were established approximately 10–36 years ago, as inferred from tree–ring analysis at breast height. Since establishment, the plantations have remained under minimal management without major silvicultural interventions (no fertilization, thinning, or other afforestation interventions). Three forest age gradients were selected for each tree species, and the site conditions were similar. The information of sites was shown in Table 1. Tree ages were confirmed by using an increment borer. Soil type data was sourced from the Soil Information Service Platform (http://www.soilinfo.cn/map/index.aspx). The soil at the study site was classified as Hapli–Ustic Cambosols according to the Chinese Soil Taxonomy (CST), corresponding to Haplic Cambisols (Ustic) in the World Reference Base for Soil Resources (WRB 2022). After removing the litter layer, soil samples were collected around the tree trunk using a soil drill with a length of 30 cm and an inner diameter of 4 cm. Three cores were taken from each tree trunk, stratified by depth (0–15 cm for the surface soil and 15–30 cm for the deep soil), and then composited into individual samples for each soil layer. Four replicates were set for each sample plot. An equal amount of fresh soil was immediately placed into sterile bags, sealed to prevent contamination, placed in foam boxes with ice cubes, transported to the laboratory, stored at −20°C in a refrigerator, and used for high–throughput sequencing to analyze soil microbial communities. The soil samples were sieved through a 2 mm mesh in small portions multiple times, and fine roots were carefully picked out from each soil layer using tweezers. The specific methods for collecting root systems are detailed in Section 2.2. A portion of the soil samples was air–dried at room temperature for the determination of soil physical and chemical properties. The remaining soil samples were stored in a refrigerator at −20°C.

**TABLE 1:** The information of study sites.

Tree species	Abbreviation	Forest age/a	Geographical position	Canopy density/%	Mean DBH/cm	Mean height/m	Forest density/trees ha^−1^
*Populus tomentosa*	PT	10	37.49° N, 114.38° E	10	6.5	4.6	1037
		16	37.48° N, 114.38° E	20	10.5	7.2	1365
		30	37.50° N, 114.39° E	30	22.3	13.4	1476
*Platycladus orientalis*	PO	12	37.49° N, 114.39° E	10	2.4	2.5	2381
		19	37.50° N, 114.39° E	60	6.8	5.5	4707
		36	37.52° N, 114.39° E	80	18.1	7.2	1659
*Styphnolobium japonicum*	SJ	9	37.45° N, 114.41° E	10	4.0	3.7	4062
		13	37.45° N, 114.41° E	40	10.1	7.1	2973
		16	37.51° N, 114.39° E	60	12.5	9.5	1111

*Note:* DBH means diameter at breast height (cm).

### 2.2 Determinations of Soil Properties and Root Traits

Soil water content (WC, %), bulk density (BD, g cm^−3^), and porosity (SP, %) were measured using the cutting–ring method. Soil conductivity (EC, μS cm^−1^) was measured with a DDS–11A conductivity meter. The soil pH was measured by the electrode potential method. The content of total organic carbon (TOC) and total nitrogen (TN, g kg^−1^) of soil was measured by an elemental analyzer (EA3000, Eurovector, Pavia, Italy). The content of total phosphorus of soil (TP, g kg^−1^) was measured by sodium hydroxide alkali melting molybdenum antimony colorimetric method, referring to LY/T 1232–2015. Specifically, 0.2 g of air–dried soil (< 0.15 mm) was fused with 2 g NaOH at high temperature, then acid–washed, diluted, and filtered. The filtrate was reacted with molybdenum–antimony–ascorbic acid reagent, and absorbance was read at 700 nm using a spectrophotometer, with TP concentration calculated from a standard curve. The soil particle size was determined by a laser particle size analyzer (Malvern Mastersizer–3000, UK), and the particle size classification standard was based on international classification standards.

The collected soil samples were passed through a 2 mm sieve. Roots remaining on the sieve were collected into self–sealing bags, while the soil that passed through was spread on a white tray, and fine roots were carefully picked out using tweezers. This process was repeated multiple times in small portions. Fine roots were washed with distilled water and scanned using an Epson Expression 10,000 XL benchtop scanner at a resolution of 300 dpi. Root morphological characteristics were analyzed using WinRHIZO software (Regent Instruments, Quebec City, QC, Canada) for image acquisition. The root length density (RLD, km m^−3^) is the total root length per unit soil volume. The root surface area density (RAD, m^2^ m^−3^) is the total root surface area per unit soil volume. The specific root length (SRL, m g^−1^) and specific root surface area (SRA, m^2^ g^−1^) are calculated as the total root length and surface area divided by dry root mass. The root tissue density (RTD, g cm^−3^) is the ratio of root dry weight to root volume. Dry roots were ground into powder to determine the carbon (RCC, %) and nitrogen content (RNC, %) using an elemental analyzer (EA3000, Euro vector, Pavia, Italy).

### 2.3 DNA Extraction and Sequencing

After extracting the soil microbial DNA by using the E.Z.N.A. soil DNA Kit (Omega Bio–tek, Norcross, GA, U.S.) in accordance with the instructions, the concentration and purity of the extracted genomic DNA were detected by 1% agarose gel electrophoresis. The primers of 515F (5′–GTGCCCAGCGG–3′) and 907R (5′–CCGTCAATTCMTTRAGTT–3′) were selected to amplify the V4–V5 regions of the bacterial 16S rRNA gene by using polymerase chain reaction (PCR) technology with the PCR instrument (ABI GeneAMP 9700 type). The ITSIF (5′–GTTGTCATTTAGGAGAGATA–3′) and ITS2R (5′–GCTGTGTTCTATCGATGC–3′) were selected as the primers for amplifying the fungal ITS1 region. The PCR products were eluted with Tris–HCl, detected by 2% agarose gel electrophoresis, and purified using the AxyPrep DNA Gel Extraction Kit (Axygen Biosciences, Union City, CA, USA). Afterwards, the purified amplified fragments were used to construct a library according to the standard operating procedures of the Illumina MiSeq platform (Illumina, PE300, San Diego, USA). The sequencing work of soil bacteria and fungi was entrusted to Shanghai Meji Biomedical Technology Co. LTD.

### 2.4 Statistical Analysis

The original sequences were utilized QIIME system and FLASH software to perform quality control, filtering, and concatenation on the original sequence. After amplifying the 16S rRNA gene of bacteria, a total of 444,995 original sequences were obtained from 72 soil samples, and 177,941 high–quality sequences were retained after quality control, with an average sequence length of 376 bp. After amplifying the fungal ITS1 region, a total of 7,023,166 original sequences were obtained, and 6,682,653 high–quality sequences were retained after quality control, with an average sequence length of 243 bp. The cluster_otus command performs 97% OTU clustering using the UPARSE–OTU algorithm. In order to obtain the species classification information corresponding to each OTU, the RDP classifier Bayesian algorithm (Wang et al. 2007) with the classification confidence level of 0.7 was used to perform taxonomic analysis on the representative sequences of OTUs with 97% similarity level, and the community species composition of each sample was statistically analyzed at each taxonomic level. Bacteria and fungi were respectively classified based on the Silva138 and Unite8.0 databases.

A one–way analysis of variance (ANOVA) and Multifactorial ANOVA were employed in SPSS (v.27.0, SPSS Inc., Chicago, USA). Microbial community structure was evaluated through Nonmetric Multidimensional Scaling (NMDS) based on the Bray‐Curtis distance metric. NMDS analysis and mapping were conducted using R (version 3.3.1) and vegan software package (version 2.4.3). Variance inflation factor (VIF) analysis was used to screen out highly autocorrelated environmental variables and select representative factors. Redundancy analysis (RDA) based on the vegan package in R studio (version 4.3.3) was used to assess the effects of soil properties and root traits on microbial community composition, with variance partitioning analysis quantifying their contribution rates. Hierarchical segmentation analysis was used to analyze the relative importance of soil properties and root traits on the Shannon index of bacteria and fungi based on the glmm.hp package in R software. The Spearman correlation heat map was plotted in Origin 2021 software.

## 3 Results

### 3.1 Soil Properties and Root Traits in Different Forest Ages and Soil Depths

In this study, the coefficients of variation of soil WC, SP, EC, TN, TOC, TP, and particle size composition were relatively large, indicating strong spatiotemporal heterogeneity of these indicators (Table S2). With advancing forest age, the TN, TOC, clay, and silt content in three species exhibited an upward trend (Table 2, Table S1); the correlation coefficients with forest age were 0.518, 0.565, 0.338, and 0.309, respectively (Table S3). The WC of *P. tomentosa* and *P. orientalis* plantations rose while BD declined with increasing forest age (Table 2 and Table S1). The WC and BD of *S. japonicum* initially increased before decreasing, whereas soil pH gradually rose with increasing forest age (Table S1). Additionally, TOC, TN, and TP decreased with soil depth.

**TABLE 2:** Multivariate ANOVA for the influences of species, forest age, soil depth, and their interactions on soil properties and root traits.

	Variable	Species	Age	Depth	Species × age	Species × depth	Age × depth	Species × age × depth
Soil	WC	**< 0.001**	**< 0.001**	**0.004**	**< 0.001**	0.072	**0.009**	0.119
	BD	**< 0.001**	**< 0.001**	0.605	**< 0.001**	0.460	0.874	**0.011**
	SP	**< 0.001**	**< 0.001**	0.078	0.212	0.137	0.997	0.273
	pH	**< 0.001**	**< 0.001**	0.522	**< 0.001**	0.498	0.276	0.560
	EC	**< 0.001**	**< 0.001**	0.761	**< 0.001**	0.981	0.123	0.744
	TOC	0.975	**< 0.001**	**< 0.001**	0.074	0.522	0.066	0.498
	TN	0.676	**< 0.001**	**< 0.001**	0.831	0.349	0.019	0.955
	TP	**< 0.001**	**0.003**	0.449	**< 0.001**	0.904	0.898	0.994
	Clay	**< 0.001**	**< 0.001**	0.903	**< 0.001**	0.738	0.219	0.611
	Silt	**< 0.001**	**< 0.001**	0.618	**< 0.001**	0.768	0.906	0.436
	Sand	**< 0.001**	**< 0.001**	0.665	**< 0.001**	0.864	0.930	0.468
Root	RB	0.976	0.724	0.066	**< 0.001**	0.230	0.588	**0.049**
	RD	0.068	0.088	**< 0.001**	**< 0.001**	0.279	0.913	0.151
	RLD	**< 0.001**	**< 0.001**	**< 0.001**	**< 0.001**	**0.022**	0.095	**< 0.001**
	RAD	**0.009**	**0.012**	0.171	**< 0.001**	**0.023**	0.875	**< 0.001**
	SRL	0.849	**0.031**	**< 0.001**	**0.005**	0.421	0.126	0.163
	SRA	0.104	0.102	**< 0.001**	**0.003**	0.939	0.233	0.199
	RTD	0.284	0.189	**0.002**	0.108	0.979	0.153	0.062
	RCC	**0.044**	**< 0.001**	0.143	**0.003**	0.381	0.054	0.439
	RNC	**< 0.001**	**< 0.001**	**< 0.001**	**< 0.001**	0.871	0.083	0.391

*Note:* The table displays the significance *p*–value. WC refers to soil water content (%); BD refers to soil bulk density (g cm^−3^); SP refers to soil porosity (%); pH refers to soil pH value; EC refers to soil conductivity (μS cm^−1^); TOC refers to soil organic carbon content (g kg^−1^); TN refers to soil total nitrogen content (g kg^−1^); TP refers to soil total phosphorus content (g kg^−1^); clay refers to the content of soil clay (%); silt refers to the content of soil silt (%); sand refers to the content of soil sand (%). RB refers to the root biomass (kg m^−3^); RD refers to root average diameter (mm); RLD refers to the root length density (km m^−3^); RAD refers to the root surface area density (m^2^ m^−3^); SRL refers to specific root length (m g^−1^); SRA refers to specific root surface area (cm^2^ mg^−1^); RTD refers to root tissue density (g cm^−3^); RCC refers to root carbon content (%), and RNC refers to root nitrogen content (%). Values in the table represent *P*‐values from the multivariate ANOVA, bold values indicate statistical significance (*P* < 0.05).

**FIGURE 1:**
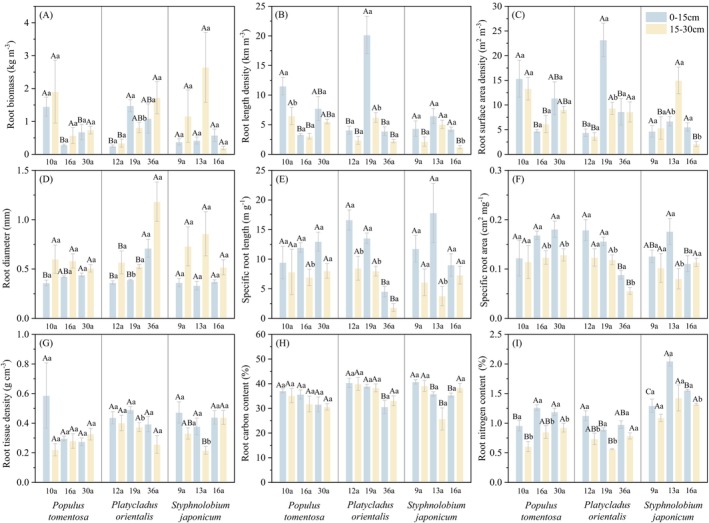
Root traits of three species in different forest ages and soil depths. Uppercase letters indicate significant differences across forest ages, and lowercase letters denote significant variations between soil depths (*p* < 0.05).

Root traits varied across species with forest age and soil depth (Figure 1; Table 2 and Table S2). The SRL, SRA, RTD, and RCC of *P. orientalis* decreased, while RB and RD gradually increased with forest age (Figure 1). The RD, SRL, SRA, and RTD of *P. tomentosa* and *S. japonicum* showed no significant changes with forest age (Figure 1). RLD, SRL, SRA, RTD, and RNC declined with soil depth (Figure 1).

### 3.2 Alpha Diversity and Composition of Soil Microbial Communities at Different Forest Ages and Soil Depths

**FIGURE 2:**
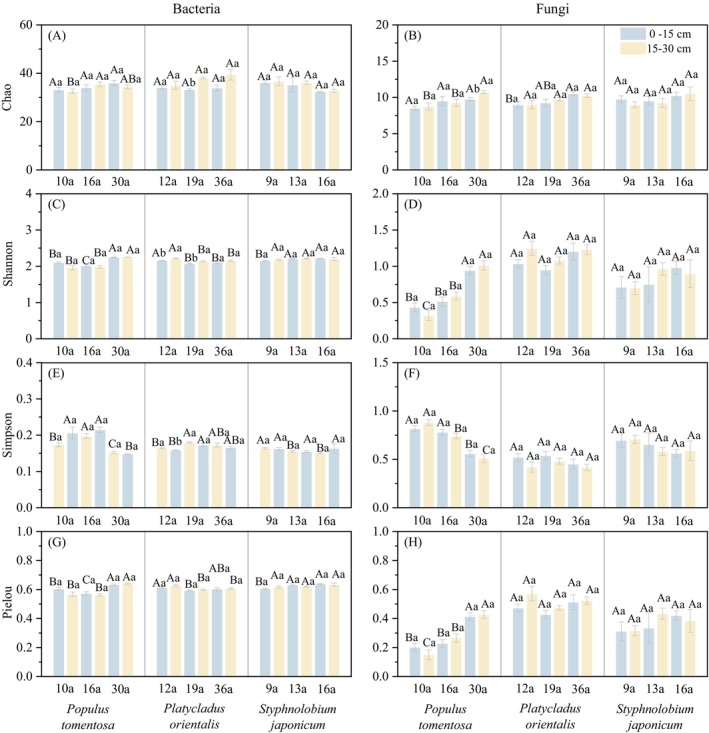
Alpha diversity index of soil bacterial and fungal communities. Uppercase letters indicate significant differences across forest ages, and lowercase letters denote significant variations between soil depths (*p* < 0.05).

The variation patterns of Shannon and Pielou_e indices were consistent, but opposite to Simpson index. The Chao, Shannon, and Pielou_e indices of soil bacteria in *P. tomentosa* plantations increased with forest age, while no significant changes were observed in *S. japonicum* plantations (Figure 2). The Shannon index of bacteria in *P. orientalis* soils decreased with forest age (Figure 2). The Chao, Shannon, and Pielou_e indices of soil fungal community increased with forest age (Figure 2 and Figure S1). The Chao index of fungi was significantly lower than that of bacteria (Figure 2 and Figure S1).

**FIGURE 3:**
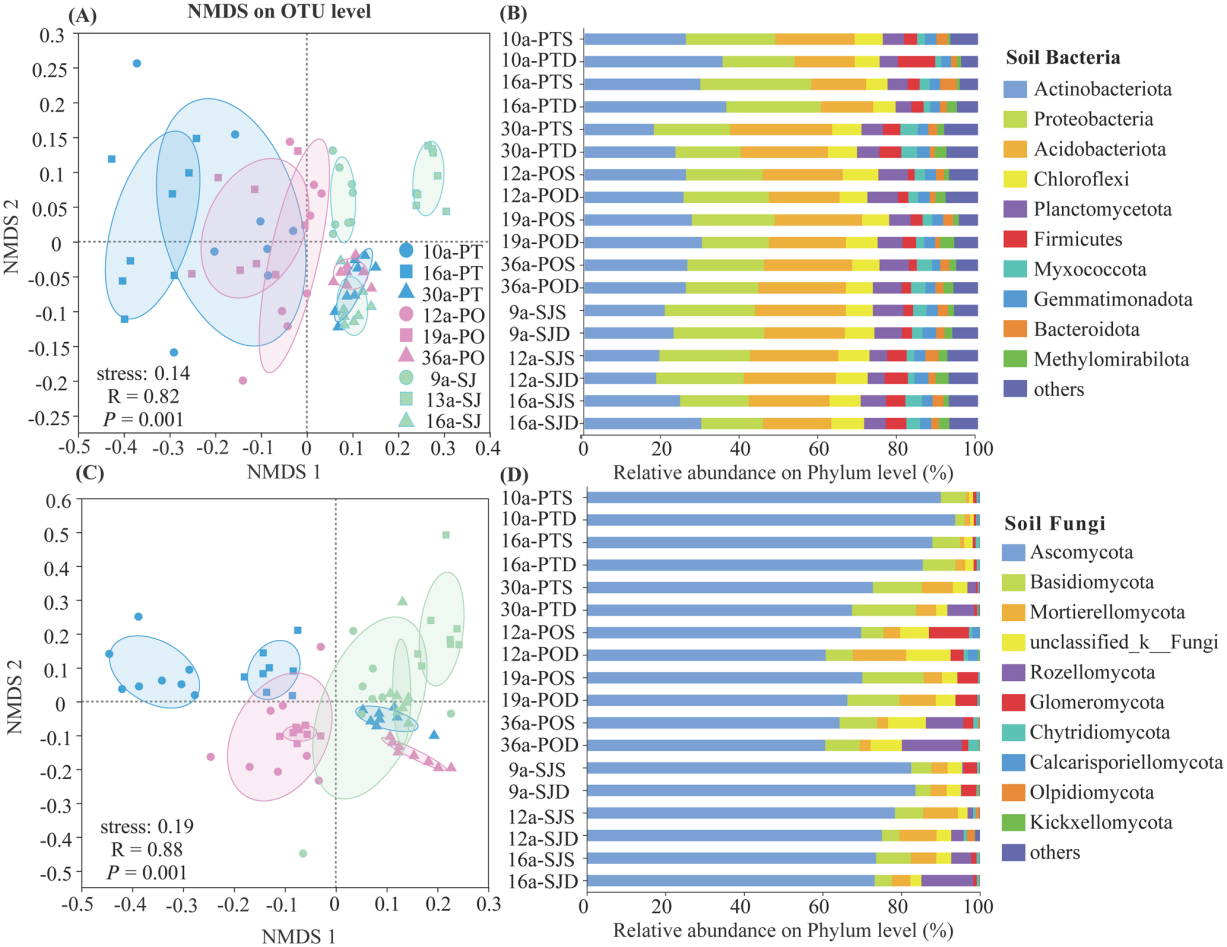
NMDS analysis of soil bacterial (A) and fungal (C) community structure at the phylum level; Bar chart of relative abundance of bacterial (B) and fungal (D) community composition. PT refers to *Populus tomentosa* plantations, PO refers to *Platycladus orientalis* plantations, and SJ refers to *Styphnolobium japonicum* plantations. The abbreviations of PTS and PTD represent surface soil and deep soil of *P. tomentosa* plantations, respectively; POS and POD represent surface soil and deep soil of *P. orientalis* plantations, respectively; SJS and SJD represent surface soil and deep soil of *S. japonicum* plantations, respectively.

The bacterial community abundance varied significantly with forest age and soil depth (Figure 3 and Figure S2; Table S4). The composition of bacterial communities in soils of 16–36a plantations showed a significant separation from that of 9–12a and 13–19a plantations across three species (Figure 3A). The composition of fungal communities in soils of *P. tomentosa* plantations showed a significant separation among forest ages (Figure 3C).

The top five soil bacterial phyla by relative abundance were Actinobacteriota (18.63%–32.74%), Proteobacteria (16.60%–26.14%), Acidobacteriota (13.64%–24.03%), Chloroflexi (5.55%–8.13%), and Planctomycetota (4.41%–7.64%, Figure 3B). The relative abundance of Actinobacteriota was lower in 30a *P. tomentosa* and 36a *P. orientalis* plantations compared with 10a and 16a *P. tomentosa* , and 12a and 19a *P. orientalis* plantations (Figure 3B).

The top five soil fungal phyla by relative abundance were Ascomycota (62.48%–91.91%), Basidiomycota (4.40%–14.43%), Mortierellomycota (1.11%–9.11%), unclassified_k_Fungi (0.95%–9.31%), and Rozellomycota (0%–12.41%, Figure 3D). With advancing forest age, the relative abundance of Ascomycota in soils of *P. tomentosa* and *S. japonicum* plantations decreased, while Basidiomycota, Rozellomycota, and Mortierellomycota increased (Figure 3D). The relative abundance of Glomeromycota and Calcarisporiellomycota gradually decreased with increasing forest age (Figure 3D and Figure S3).

### 3.3 Relationships Between Soil Properties, Root Traits, and Alpha Diversity of Soil Microbial Communities

**FIGURE 4:**
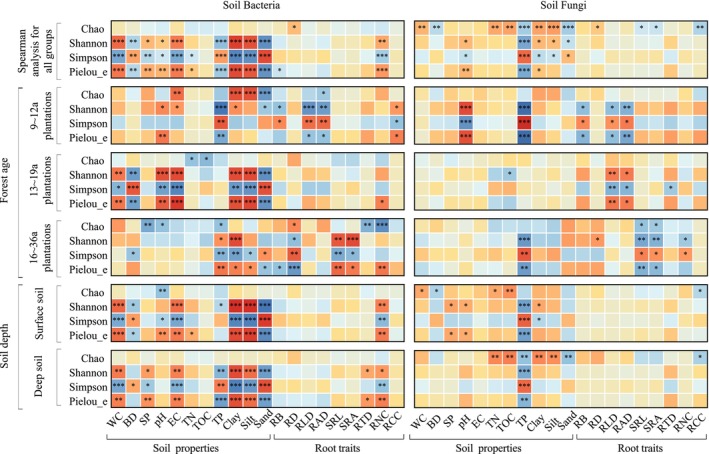
Correlation analysis between Alpha diversity indices and factors (soil properties and root traits). The significant effects were reported at *p* < 0.05 (*), *p* < 0.01 (**) or *p* < 0.001 (***) levels. The explanations of abbreviations are explained behind Table 2.

Clay, silt, WC, and pH were positively correlated with microbial diversity, while TP was the opposite (Figure 4). Soil bacterial diversity in 9–12a and 16–36a plantations was closely linked to soil properties and root traits (Figure 4). In 13–19a plantations, bacterial diversity showed significant correlations with soil properties (Figure 4), while fungi showed significant correlations with root traits (Figure 4). Soil properties were more correlated with microbial Alpha diversity than root traits (Figure 4).

**FIGURE 5:**
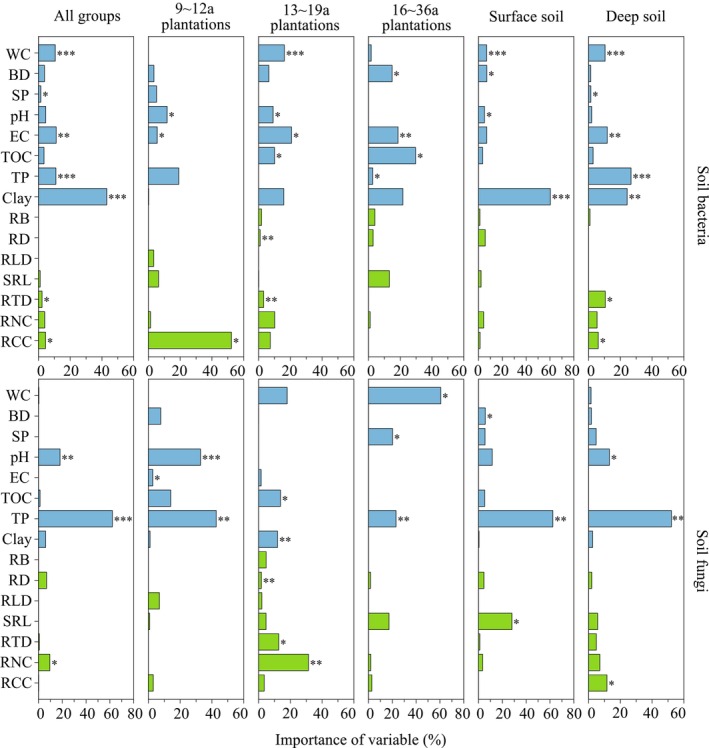
The relative importance ranking of soil properties and root traits on the Shannon index. Indicators with negative values of relative importance are not displayed. The significant effects were reported at *p* < 0.05 (*), *p* < 0.01 (**), or *p* < 0.001 (***) levels. The explanations of abbreviations are explained behind Table 2.

Hierarchical segmentation analysis showed that soil properties affecting the Shannon index of soil bacteria were soil clay (relative importance of 43.04%), EC (11.18%), TP (11.02%), and WC (10.58%) (*p* < 0.05; Figure 5). The root traits affecting bacterial diversity were mainly RCC (4.48%) and RTD (2.29%) (*p* < 0.05; Figure 5). In 9–12a plantations, soil bacteria diversity was mainly affected by RCC (61.40%), pH (11.75%), and soil EC (5.51%). Bacterial diversity in 13–19a plantations was mainly influenced by EC (20.76%), WC (16.23%), TOC (10.05%), pH (9.16%), and RTD (3.16%), while that in 16–36a plantations was mainly influenced by soil TOC (29.71%), EC (18.61%), BD (14.90%), and TP (2.69%) (*p* < 0.05; Figure 5).

Soil properties and root traits had a greater impact on soil bacterial diversity than fungal diversity (Figures 4 and 5). The primary factors influencing fungal diversity were soil TP (62.13%), pH (18.15%), and RNC (9.60%) (*p* < 0.05; Figure 5). In 9–12a and 16–36a plantations, fungal diversity was mainly driven by soil properties (WC, SP, pH, and TP) rather than root traits. In 13–19a plantations, fungal diversity was strongly influenced by soil properties (TOC of 13.81% and clay of 11.97%) and root traits (RNC of 31.46% and RTD of 12.74%) (*p* < 0.05; Figure 5).

### 3.4 Relationships Among Relative Abundances of Soil Microbial Community, Soil Properties, and Root Traits

**FIGURE 6:**
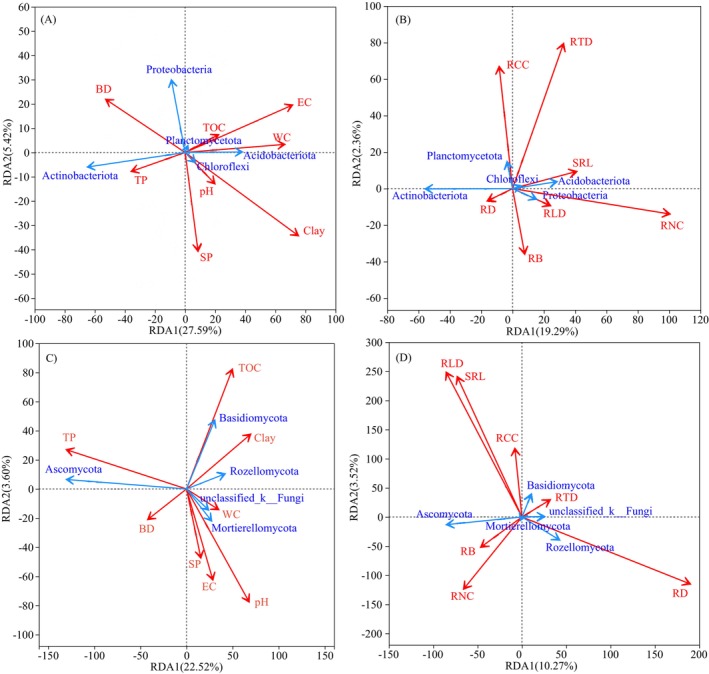
RDA analysis of the impacts of soil properties (A, C) and root traits (B, D) on bacteria and fungi. The explanations of abbreviations are explained behind Table 2.

The relative abundance of Actinobacteriota was markedly negatively associated with soil WC, EC, clay, and silt (*p* < 0.001), but positively linked to soil BD and sand (*p* < 0.05; Figure 6 and Figure S4). The Acidobacteriota showed the opposite trend to Actinobacteriota (Figure 6 and Figure S4). Ascomycota was markedly positively linked to soil TP and sand, while markedly negatively associated with clay, silt, and pH. Basidiomycota was markedly negatively associated with soil EC, while markedly positively linked to TN, TOC, and RTD (*p* < 0.05; Figure 6 and Figure S4).

**FIGURE 7:**
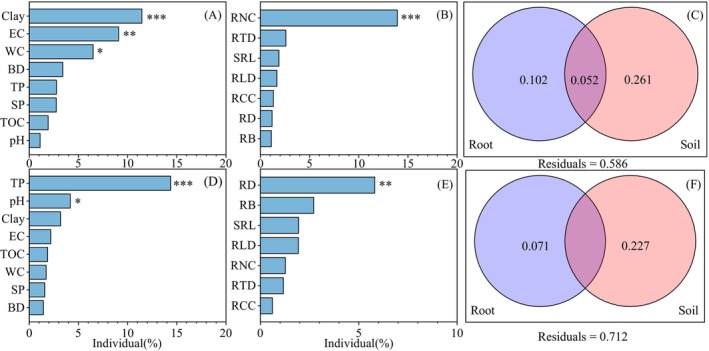
The impacts of soil properties (A) and root traits (B) on bacterial abundance, and soil properties (D) and root traits (E) on fungal abundance. Venn diagrams of variance partitioning analysis for explanatory rates of soil and root for (C) bacterial and (F) fungal communities. The explanations of abbreviations are explained behind Table 2.

Redundancy analysis showed that soil factors affecting the composition of bacterial communities were soil clay (individual effects of 11.46%), EC (9.09%), and WC (6.49%) (Figures 6A and 7A). The TP (14.37%), pH (4.18%), and clay (3.18%) were primary contributors to fungal communities (Figures 6C and 7D). Root traits including RNC (13.92%) and RD (5.81%) exerted significant influences on bacteria and fungi, respectively (Figures 6 and 7). Variance partitioning analysis showed that soil properties and root traits explained 41.4% of bacterial composition change and 28.8% of fungal composition change (Figure 7C,F). Soil properties explained 26.10% of bacterial and 22.70% of fungal community changes (Figure 7C), while root traits explained 10.20% of bacterial and 7.10% of fungal community changes (Figure 7F).

## 4 Discussion

### 4.1 Changes of Soil Properties and Root Traits With Forest Age and Soil Depth

The soil water content of *P. tomentosa* and *P. orientalis* plantations showed a gradual increase with forest age. This could result from the accumulation of litter, which limits direct sunlight exposure, decreases soil water evaporation (Guo et al. 2024). The soil BD decreased significantly while the SP increased with forest age, soil loosening, and aggregate formation due to root growth, ultimately enhancing soil permeability. The content of soil nutrients exhibited a decreasing trend with increasing soil depth, which aligned with previous studies (Wu et al. 2020), and supported part of our first hypothesis. Microbial degradation promotes the formation and accumulation of soil organic matter (Emiru and Gebrekidan 2013), thereby increasing the thickness of surface humus and improving soil fertility. On the other hand, microbial respiration also leads to the loss of some organic carbon, but microbial respiratory activities decline rapidly with the increase in soil depth (Fang and Moncrieff 2005). Therefore, the higher nutrient content in surface soil compared to deeper soil may result from a combination of factors, including greater inputs of litter and fine roots, as well as higher microbial activity.

In this study, changes in root biomass (RB) with forest age varied across tree species. Root biomass of *P. orientalis* plantations increased with forest age, similar to *Pinus strobus* plantations (Peichl and Arain 2006). However, 10a *P. tomentosa* plantations had the highest RB possibly because they developed more root biomass to acquire more water and nutrients in the early growth stages (Claus and George 2005). The SRL and SRA decreased, and RD increased with forest age in *P. orientalis* plantations, which was similar to the results of *Larix principis–rupprechtii* (Liu, Chen, and Chen 2024) and *Fraxinus mandshurica* (Li et al. 2021) plantations. Younger‐aged plantations need to develop more effective root systems to cope with survival pressures such as limited soil resources (Jagodzinski et al. 2016). In addition, the SRL and SRA of surface soil roots were higher than those of deep soil, and the RD was lower than that of deep soil, which supported part of our first hypothesis. Plants could adjust their root morphology to utilize resources at different soil depths (Peng and Chen 2021). This supports our first scientific hypothesis. Due to the fact that nutrients were more abundant in surface soil than deep soil, plants enhanced nutrient absorption from surface soil by increasing specific root length and other traits.

### 4.2 Diversity and Composition of Soil Bacterial and Fungal Communities With Forest Age and Soil Depth

In this study, the Alpha diversity of bacteria varied with forest age depending on tree species. The Chao, Shannon, and Pielou_e indices of soil bacteria in *P. tomentosa* plantations increased with forest age, while no significant changes were observed in *S. japonicum* plantations (Figure 2). These changing trends indicate that the influence of forest age on bacterial communities is not uniform but rather specific to tree species. This confirmed the results of previous studies that bacterial diversity showed an increasing trend in *Eucalyptus urograndis* plantations (Xu et al. 2020) and a decreasing trend in *Populus euramevicana* plantations (Liu, Hou, et al. 2024) with forest age. This difference among tree species indicates that specific factors of trees, such as the mass of root secretions or litter, may have different effects on bacterial communities (Yuan and Chen 2012). In addition, Marschner et al. (2004) reported that three plant species (chickpea, canola, Sudan grass) had distinct rhizosphere microbial communities, indicating that the plant itself exerted a highly selective effect on the rhizosphere microbe. In addition to the differences in root secretions, the variations in bacterial Alpha diversity among different forest ages and tree species can also be attributed to the differences in litter and root traits and the long‐term changes in soil physical and chemical properties (Prescott and Grayston 2013). In addition, tree species can alter the microclimate conditions under the forest (Ao et al. 2022), indirectly influencing the pattern of soil microbial diversity. In contrast, the soil fungal diversity indices (Chao, Shannon, and Pielou_e) increased with forest age, which supported part of our second hypothesis. Compared with bacteria, fungi exhibited a lower Chao index, making them more sensitive to resource input changes associated with stand development. One possible explanation is that bacteria preferentially utilize simple and easily accessible carbon sources (Dang et al. 2018), which enables them to maintain relatively stable communities. In contrast, fungi rely on more complex nutrient resources (Niu et al. 2015), making them more sensitive to resource input changes associated with stand development. Consistently, the coefficients of variation (CV) of fungal community composition were generally higher than those of bacteria, further indicating that fungal communities were more variable and susceptible to environmental changes.

The composition of soil bacterial communities in 16–36a plantations showed significant separation compared to 9–12a and 13–19a plantations, indicating that soil bacteria and fungi underwent succession with forest age. At the phylum level, the relative abundance of Actinobacteriota was the highest. Actinobacteriota are a unique group of bacteria characterized by their tolerance to drought and heavy metals, as well as their remarkable halophilic and alkaliphilic properties, which enable them to survive in extreme environments (Cui et al. 2023). For instance, Actinobacteriota includes genera such as Streptomyces, Micrococcus, Rhodococcus, Geodermatophilus, and Citricoccus, all of which can survive in extremely arid environments (Okoro et al. 2009). Moreover, Streptomyces possesses remarkable tolerance to heavy (and related) metals (Farda et al. 2022). The Actinobacteriota exhibit strong environmental adaptability, which explains their relatively high abundance. Certain Actinobacteriota groups secrete cellulase, chitinase, and peroxidase to break down complex organic matter (Shivlata and Satyanarayana 2017), including lignin and cellulose (Pankratov et al. 2011). However, in 30a *P. tomentosa* and 36a *P. orientalis* plantations, the relative abundance of Actinobacteria decreased, which may be due to substrate availability or microbial competition. The predominant fungal phyla in the soil were Ascomycota, Basidiomycota, and Mortierellomycota. This was similar to previous research that Ascomycota and Basidiomycota occupied a major advantage in forest ecosystems (Huang et al. 2023), which could effectively degrade cellulose in litters to maintain their own nutrition and improve soil fertility (Geethanjali and Jayashankar 2016). We found that the relative abundance of Ascomycota declined with forest age in *P. tomentosa* and *S. japonicum* plantations, whereas Basidiomycota increased. The possible reason was that Ascomycota evolved faster reproductive rates and greater radiation tolerance than Basidiomycota (Rainey et al. 2005), making them more suitable for living in soils of younger‐aged plantations with lower canopy closure than those in older‐aged plantations. Moreover, the higher CV of fungal community composition compared with bacteria suggested that fungal communities responded more dynamically to forest age changes.

Earlier research indicated that Alpha diversity of microbiota generally declined with increasing soil depth (Du et al. 2017). Contradicting our second scientific hypothesis, the variation of microbial Alpha diversity between soil depths was not significant. Soils with higher organic carbon had higher microbial diversity than soil lacking organic carbon (Tian et al. 2021; Hao et al. 2021). Even though the organic carbon content in the surface soil was significantly higher than that in the deep soil, the bacterial richness index had no differences between soil depths. This may be related to the carbon fractions, and it was available carbon rather than total carbon content that affected microbial richness and diversity (Eilers et al. 2012). The relative abundance of fungi in 0–15 cm soil typically exceeded that in 15–30 cm soil. This may be because the rich nutrients, good air permeability, and abundant oxygen of the surface soil can provide favorable conditions for fungi (Chen et al. 2021). On the other hand, Firmicutes in bacteria exhibited the opposite pattern, which may be due to the fact that Firmicutes are chemodynamic heterotrophic bacteria that do not require high light and were more active in deep soils (Bai et al. 2025).

### 4.3 Driving Factors for Composition and Diversity of Soil Microbial Community

Unexpectedly, our findings revealed that soil properties had a more pronounced effect on the composition and diversity of the microbial community than root traits. This was contrary to our third scientific hypothesis. In this study, soil WC played a crucial role in influencing the composition and diversity of microbial communities. Moisture alters soil microbial activity and community structure in multiple ways, including by influencing soil properties, organic matter availability, and diffusion and transport capacity (Zhu et al. 2019). When the soil moisture content is low, soil respiration weakens, and the metabolic activity of microorganisms and the diffusion of respiratory substrates are restricted, which inhibits the efficiency of soil microorganisms in obtaining nutrients. When the moisture content is too high, it restricts the diffusion of oxygen and affects the oxygen‐consuming respiration of microorganisms, thereby suppressing the activity of soil microorganisms (Zhu et al. 2019; Li et al. 2019). Soil WC showed positive associations with microbial diversity, particularly bacterial diversity. This similar result has been observed in wheat farmland soil (Banerjee et al. 2016), temperate grassland (Zhai et al. 2024), forest, and shrubland soils (He et al. 2023). Increasing soil water content enhanced the rate of liquid diffusion, providing carbon and nitrogen substrates for microorganisms, which were key factors in building microbial communities and activity. In addition, soil texture has a significant impact on the composition of soil bacterial and fungal communities. We observed a strong positive relationship between soil clay and silt content and Alpha diversity of bacteria and fungi, and the influence of clay on diversity of bacteria (43.04%) was greater than fungi (6.00%). This was similar to previous evidence that there was no demonstrable link between microbial diversity and sand particles (Poll et al. 2003). This may be due to the low nutrient content, low cation exchange capacity, and adsorption capacity of sand particles compared to clay and silt (Roberts 2004). At the same time, the limited pore connectivity of clay promoted bacterial diversity in soil (Carson et al. 2010). The relationship between microbial diversity and clay aligned with earlier findings, which showed that soil clay content was positively associated with bacterial diversity but not fungal diversity in arable lands (Naveed et al. 2016; Seaton et al. 2020). Bacterial activity and communities are mainly confined to areas filled with water and can only move very short distances due to their limiting directed movement. However, fungi are much less restricted to areas of hydration, and they rely on mycelium to get nutrients from vast distances to survive. Therefore, the bacterial colonies were more strongly affected by silt and clay with smaller particle sizes when the moisture was appropriate.

Conductivity demonstrated the degree of salt in the soil, and high conductivity usually meant higher salt content. Fu et al. (2012) found that soil salinity concentration showed a significant inverse relationship with microbial diversity. On the other hand, certain bacteria may be more adapted to high salt environments, thus gaining an advantage in soils with high conductivity. The soil TP was another key factor determining soil microbial communities. For example, Liu et al. (2018) found that phosphorus content was significantly negatively correlated with bacterial and fungal Alpha indices. We found that soil TP had a stronger negative effect on fungi than on bacteria. Soil fungi are oligotrophic, and they have lower nutrient requirements, a slower growing rate, and higher carbon use efficiency compared to bacteria (Leff et al. 2015). Ma et al. (2022) found that soil fungi were more susceptible to nutrient inputs than bacteria, and phosphorus addition significantly reduced fungal richness and changed fungal community composition rather than soil bacteria. The possible reason is that in nitrogen–limited forests, phosphorus addition increases the imbalance of soil nitrogen and phosphorus, thus reducing fungal abundance. In another way, higher soil phosphorus levels may reduce plant reliance on mycorrhizal fungi, thereby decreasing carbon allocation to these fungi (Johnson et al. 2008; Yu et al. 2022). This was supported by evidence that Glomeromycota phylum decreased with increased soil phosphorus (Figure S4), which were mycorrhizal.

Root of plants acts as a link for plant–soil interactions, and its traits play an important role in regulating soil microbial communities (Liu et al. 2022) and influencing nutrient cycling (Bardgett et al. 2014). The effect of soil properties on bacterial diversity increased, while root effects gradually decreased with increasing forest age. This supported our third hypothesis. It was speculated that in the early stage of planting, the growth rate of younger–aged plantations was relatively fast, and the changes in root exudates and root traits caused frequent interactions between roots and soil, which greatly affected soil microbiota. As forest succession progresses, the adaptation and selection of root and soil tended to be stable. In addition, litter gradually accumulated in the surface soil with forest age, and climate changes such as precipitation made soil properties more susceptible to change. Therefore, soil microbiota was more affected by soil properties in older–aged plantations. As the soil depth increased, the influence of root traits on bacterial Shannon index was more significant than that on fungal. The SRL and soil TP were the primary drivers influencing fungal diversity in the surface soil. The root with higher activity, higher SRL, and lower RD in the surface soil than that in deep soil (Tückmantel et al. 2017) contributed to enhancing fungal diversity and altering community composition.

In line with our expectations, RCC, RNC, RTD, and SRL exerted beneficial effects on microbial community structure and diversity. Earlier research indicated that root exudates, RNC served as essential nutrient sources for soil microorganisms (Kramer et al. 2010). RL and RB affected the release of root exudates, which subsequently affect soil microbial communities (Saleem et al. 2018). In addition, tree species with rapid acquisition strategies were accompanied by higher microbial diversity (Gillespie et al. 2021), which had higher RNC and RCC. The influence of soil properties and root traits on soil bacterial composition and diversity was greater than that on fungi in this study, which was consistent with a previous study that root nitrogen content influenced bacterial diversity but not fungal (Merino‐Martín et al. 2020). This may be due to the fact that compared to fungi with hyphal growth, the transmission ability was limited for bacteria. In addition, Brant et al. (2006) revealed that the input of root carbon played a greater role in controlling microbial community composition compared to the input of litter.

## 5 Conclusion

Study revealed that the relative importance of soil properties and root traits in shaping soil microbial communities shifts with forest age and soil depth. Specifically, the influence of root traits on bacterial diversity weakened with stand development, while the influence of soil properties on bacterial diversity gradually increased. In contrast, with increasing soil depth, root traits exerted greater effects on bacteria but weaker effects on fungi. Soil and root exerted a greater impact on bacteria (41.4%) compared to fungi (28.8%). Moreover, bacterial and fungal communities were driven by different key factors, reflecting distinct ecological strategies. These results deepen our understanding of belowground plant–soil–microbe interactions, providing a basis for the management and restoration of forest ecosystems. However, our findings only reflect differences among the specific age classes sampled in this study, rather than a comprehensive representation of long–term forest succession. Future research should therefore incorporate longer chronosequences to capture continuous successional dynamics.

## Author Contributions

**Yaxuan Chen:** formal analysis (equal), investigation (equal), methodology (equal), writing – original draft (lead). **Qianyuan Liu:** formal analysis (equal), funding acquisition (lead), investigation (equal), writing – review and editing (equal). **Yanmei Chen:** funding acquisition (equal), writing – review and editing (equal). **Changqi Ai:** writing – review and editing (equal). **Peipei Jiang:** writing – review and editing (equal).

## Conflicts of Interest

The authors declare no conflicts of interest.

## Supporting information


**Data S1:** Supporting Information.

**Table S1:** Soil physicochemical properties of *Populus tomentosa* , *Platycladus orientalis* , and *Styphnolobium japonicum* plantations under different forest ages and soil depths.
**Table S2:** Variation description of soil characteristics.
**Table S3:** Correlation analysis of soil properties, root traits and forest age.
**Table S4:** Multivariate ANOVA for the effects of species, forest age, soil depth, and interactions on microbial communities and diversity indies. The table displays the significance *p*‐value.
**Figure S1:** The main effect of soil depth, forest age, and species on the Alpha diversity indies of bacterial (A, C, E, and G) and fungal (B, D, F, and H) communities on phylum level. Bars and errors show means ± SE. Bars sharing the same letter are not different between treatments at *p* < 0.05.
**Figure S2:** The main effect of soil depth, forest age, and species on the relative abundance of each bacterial species on phylum level. Bars and errors show means ± SE. Bars sharing the same letter are not different between treatments at *p* < 0.05.
**Figure S3:** The main effect of soil depth, forest age, and species on the relative abundance of each fungal species on phylum level. Bars and errors show means ± SE. Bars sharing the same letter are not different between treatments at *p* < 0.05.
**Figure S4:** Correlation analysis between the relative abundance of bacteria and fungi communities and factors (soil properties and root traits). The significant effects were reported at *p* < 0.05 (*), *p* < 0.01 (**) or *p* < 0.001 (***) levels. The abbreviation explanations of soil properties and root traits are shown in the Table 1.

## Data Availability

All the required data is uploaded as Supporting Information.
