# Supplementary material for: Forest Age and Soil Depth Mediate the Effects of Soil and Root Traits on Soil Microbial Community in Plantations
Source: Ecol Evol. 2025 Oct 14;15(10):e72264. doi: 10.1002/ece3.72264 (PMC12521610; doi:10.1002/ece3.72264)
Supplement: Supplementary file 2 — Table S1: Soil physicochemical properties of Populus tomentosa , Platycladus orientalis , and Styphnolobium japonicum plantations under different forest ages and soil depths. Table S2: Variation description of soil characteristics. Table S3: Correlation analysis of soil properties, root traits and forest age. Table S4: Multivariate ANOVA for the effects of species, forest age, soil depth, and interactions on microbial communities and diversity indies. The table displays the significance p‐value. Figure S1: The main effect of soil depth, forest age, and species on the Alpha diversity indies of bacterial (A, C, E, and G) and fungal (B, D, F, and H) communities on phylum level. Bars and errors show means ± SE. Bars sharing the same letter are not different between treatments at p < 0.05. Figure S2: The main effect of soil depth, forest age, and species on the relative abundance of each bacterial species on phylum level. Bars and errors show means ± SE. Bars sharing the same letter are not different between treatments at p < 0.05. Figure S3: The main effect of soil depth, forest age, and species on the relative abundance of each fungal species on phylum level. Bars and errors show means ± SE. Bars sharing the same letter are not different between treatments at p < 0.05. Figure S4: Correlation analysis between the relative abundance of bacteria and fungi communities and factors (soil properties and root traits). The significant effects were reported at p < 0.05 (*), p < 0.01 (**) or p < 0.001 (***) levels. The abbreviation explanations of soil properties and root traits are shown in the Table 1. [file ECE3-15-e72264-s001.docx]

**Forest age and soil depth mediate the effects of soil and root traits on soil microbial community in plantations**

| **Table S1** Soil physicochemical properties of *Populus tomentosa*, *Platycladus orientalis*, and *Styphnolobium japonicum* plantations under different forest ages and soil depths | | | | | | | | | | |
| --- | --- | --- | --- | --- | --- | --- | --- | --- | --- | --- |
| Variable | Soil Depth | *Populus tomentosa* | | | *Platycladus orientalis* | | | *Styphnolobium japonicum* | | |
|  |  | 10a | 16a | 30a | 12a | 19a | 36a | 9a | 13a | 16a |
| WC | 0-15 | 2.60±0.94 Ba | 2.83±0.22 Ba | 9.32±0.67 Aa | 2.06±0.45 Ba | 2.88±0.51 Ba | 7.96±0.55 Ab | 5.83±0.20 Bb | 19.81±0.83 Aa | 6.87±0.35 Bb |
|  | 15-30 | 2.88±0.72 Ba | 2.95±0.30 Ba | 9.16±0.65 Aa | 3.05±0.24 Ba | 2.19±0.38 Ba | 10.05±0.45 Aa | 7.12±0.33 Ca | 19.63±1.12 Aa | 10.93±0.93 Ba |
| BD | 0-15 | 1.47±0.05 Aa | 1.49±0.03 Aa | 1.22±0.04 Ba | 1.49±0.03 Aa | 1.48±0.06 Aa | 1.15±0.04 Ba | 1.18±0.02 Bb | 1.27±0.02 Aa | 1.31±0.02 Aa |
|  | 15-30 | 1.39±0.08 Aa | 1.42±0.08 Aa | 1.28±0.02 Aa | 1.45±0.05 Aa | 1.50±0.02 Aa | 1.25±0.04 Ba | 1.29±0.03 Aa | 1.24±0.03 ABa | 1.14±0.05 Bb |
| SP | 0-15 | 3.45±0.30 Ab | 4.31±0.38 Aa | 4.65±0.38 Aa | 6.26±0.78 ABa | 4.21±0.64 Ba | 8.33±1.11 Aa | 7.03±1.17 ABa | 4.14±1.12 Ba | 8.47±0.78 Aa |
|  | 15-30 | 5.10±0.53 ABa | 4.14±0.35 Ba | 6.58±0.69 Aa | 6.74±0.91 Aa | 4.87±1.52 Aa | 5.85±1.55 Aa | 7.61±1.43 Aa | 6.54±0.65 Aa | 11.66±2.49 Aa |
| pH | 0-15 | 7.22±0.18 Aa | 7.53±0.09 Aa | 7.36±0.07 Aa | 7.76±0.07 Aa | 7.57±0.04 Aa | 7.60±0.09 Aa | 7.46±0.05 Ca | 7.81±0.08 Ba | 8.05±0.04 Aa |
|  | 15-30 | 7.39±0.10 Aa | 7.61±0.13 Aa | 7.34±0.04 Aa | 7.68±0.07 Aa | 7.70±0.08 Aa | 7.65±0.05 Aa | 7.40±0.04 Ba | 7.92±0.07 Aa | 7.89±0.06 Aa |
| EC | 0-15 | 41.85±11.25 Aa | 36.08±2.73 Aa | 62.34±7.68 Aa | 60.53±6.56 Ba | 37.90±3.07 Ca | 79.81±2.89 Aa | 104.79±1.67 Ba | 127.28±1.05 Aa | 81.56±1.71 Cb |
|  | 15-30 | 42.51±8.93 Ba | 32.59±1.32 Ba | 69.04±4.15 Aa | 62.71±3.54 Ba | 36.09±5.52 Ca | 81.16±2.44 Aa | 104.56±0.63 Ba | 116.91±3.14 Ab | 92.81±1.47 Ca |
| TOC | 0-15 | 5.38±0.81 Ba | 8.98±1.48 Aa | 9.73±0.20 Aa | 4.98±0.58 Ba | 9.03±1.06 Aa | 10.80±0.56 Aa | 6.30±0.59 Ba | 7.65±0.18 Ba | 11.38±0.51 Aa |
|  | 15-30 | 3.53±0.29 Ba | 6.08±1.19 Aa | 6.48±0.38 Ab | 3.83±0.63 Ba | 4.10±0.86 Bb | 7.68±0.83 Ab | 3.33±0.15 Cb | 4.15±0.13 Bb | 7.05±0.43 Ab |
| TN | 0-15 | 0.38±0.03 Ba | 0.63±0.12 Aa | 0.78±0.03 Aa | 0.43±0.05 Ba | 0.60±0.13 ABa | 0.78±0.03 Aa | 0.45±0.06 Ca | 0.68±0.05 Ba | 0.88±0.03 Aa |
|  | 15-30 | 0.30±0.04 Ba | 0.38±0.09 ABa | 0.55±0.03 Ab | 0.33±0.06 Ba | 0.28±0.09 Ba | 0.58±0.03 Ab | 0.30±0.00 Ba | 0.33±0.03 Bb | 0.53±0.05 Ab |
| TP | 0-15 | 1.53±0.48Aa | 0.56±0.03Ba | 0.21±0.01Ba | 0.22±0.02Ba | 0.48±0.06Aa | 0.16±0.01Ba | 0.28±0.01Ba | 0.52±0.03Aa | 0.31±0.02Ba |
|  | 15-30 | 1.44±0.43Aa | 0.40±0.05Bb | 0.17±0.00Bb | 0.18±0.02Ba | 0.43±0.06Aa | 0.12±0.01Bb | 0.24±0.01Bb | 0.33±0.02Ab | 0.17±0.01Cb |
| Clay | 0-15 | 0.10±0.10 Ba | 0.06±0.03 Ba | 5.65±0.29 Aa | 0.29±0.06 Ba | 0.08±0.04 Ba | 3.27±0.28 Aa | 2.13±0.35 Ba | 3.41±0.50 ABa | 4.22±0.75 Aa |
|  | 15-30 | 0.08±0.08 Ba | 0.25±0.13 Ba | 5.30±0.27 Aa | 0.35±0.12 Ba | 0.11±0.07 Ba | 2.96±0.79 Aa | 3.14±0.12 Ba | 3.27±0.15 Ba | 3.92±0.23 Aa |
| Silt | 0-15 | 11.98±3.84 Ba | 12.75±1.03 Ba | 77.01±1.43 Aa | 15.78±1.48 Ba | 13.12±1.85 Ba | 64.76±2.91 Aa | 63.93±3.35 Aa | 70.86±1.97 Aa | 74.89±4.60 Aa |
|  | 15-30 | 11.76±3.43 Ba | 18.44±4.12 Ba | 77.50±1.75 Aa | 16.93±2.29 Ba | 14.29±1.90 Ba | 65.33±7.55 Aa | 65.00±2.00 Ba | 63.94±3.44 Ba | 78.68±1.70 Aa |
| Sand | 0-15 | 87.43±3.86 Aa | 86.44±0.78 Aa | 17.35±1.68 Ba | 83.64±1.36 Aa | 86.57±1.79 Aa | 31.97±3.18 Ba | 33.94±3.22 Aa | 25.74±1.71 ABa | 20.90±5.34 Ba |
|  | 15-30 | 88.12±3.49 Aa | 81.28±4.23 Aa | 17.20±1.97 Ba | 82.26±2.41 Aa | 85.09±1.82 Aa | 31.71±8.27 Ba | 31.87±2.11 Aa | 32.80±3.57 Aa | 17.40±1.71 Ba |

Note: WC refers to soil water content (%); BD refers to soil bulk density (g cm^-3^); SP refers to soil porosity (%); pH refers to soil pH value; EC refers to soil conductivity (μS cm^-1)^; TOC refers to soil organic carbon content (g kg^-1^); TN refers to soil total nitrogen content (g kg^-1^); TP refers to soil total phosphorus content (g kg^-1^); C / N refers to soil carbon-nitrogen ratio; C / P refers to soil carbon-phosphorus ratio; N / P refers to soil nitrogen-phosphorus ratio; Clay refers to the content of soil clay (%); Silt refers to the content of soil silt (%); Sand refers to the content of soil sand (%). Data were represented as the mean ± standard error (n=4). Different uppercase letters indicate significant differences during forest age, while different lowercase letters indicate significant differences between soil depth (*P* < 0.05).

**Table S2** Variation description of soil characteristics

| Group | Variable | Units | Numbers | Min | Max | Mean | Standard  deviation | Coefficient of variation |
| --- | --- | --- | --- | --- | --- | --- | --- | --- |
| Soil | WC | % | 72 | 1.17 | 21.38 | 7.12 | 5.47 | 0.77 |
|  | BD | g cm^-3^ | 72 | 1.05 | 1.65 | 1.33 | 0.15 | 0.11 |
|  | SP | % | 72 | 1.36 | 18.89 | 6.11 | 2.75 | 0.45 |
|  | pH | - | 72 | 6.73 | 8.17 | 7.61 | 0.26 | 0.03 |
|  | EC | μS cm^-1^ | 72 | 16.69 | 130.00 | 70.58 | 30.49 | 0.43 |
|  | TN | g kg^-1^ | 72 | 0.10 | 0.90 | 0.51 | 0.21 | 0.42 |
|  | TOC | g kg^-1^ | 72 | 2.30 | 12.90 | 6.69 | 2.76 | 0.41 |
|  | TP | g kg^-1^ | 72 | 1.70 | 43.09 | 7.26 | 8.08 | 1.11 |
|  | C/N | - | 72 | 9.00 | 26.00 | 13.57 | 3.00 | 0.22 |
|  | C/P | - | 72 | 0.09 | 4.67 | 1.58 | 1.15 | 0.73 |
|  | N/P | - | 72 | 0.01 | 0.35 | 0.12 | 0.09 | 0.72 |
|  | Clay | % | 72 | 0.00 | 6.47 | 2.14 | 2.02 | 0.94 |
|  | Silt | % | 72 | 5.54 | 83.68 | 45.38 | 28.87 | 0.64 |
|  | Sand | % | 72 | 12.53 | 93.88 | 52.32 | 30.59 | 0.58 |
| Root | RB | kg m^-3^ | 72 | 0.06 | 5.33 | 0.93 | 1.02 | 1.09 |
|  | RD | mm | 72 | 0.27 | 1.70 | 0.55 | 0.28 | 0.51 |
|  | RLD | km m^-3^ | 72 | 0.63 | 26.75 | 5.56 | 4.77 | 0.86 |
|  | RAD | m^2^ m^-3^ | 72 | 0.77 | 30.17 | 8.72 | 6.28 | 0.72 |
|  | SRL | m g^-1^ | 72 | 0.74 | 28.27 | 9.22 | 5.58 | 0.60 |
|  | SRA | cm^2^ mg^-1^ | 72 | 0.03 | 0.24 | 0.13 | 0.05 | 0.38 |
|  | RTD | g cm^-3^ | 72 | 0.14 | 1.04 | 0.37 | 0.15 | 0.42 |
|  | RNC | % | 72 | 0.42 | 2.23 | 1.09 | 0.39 | 0.36 |
|  | RCC | % | 72 | 15.03 | 44.87 | 35.47 | 5.62 | 0.16 |
| Soil Bacteria | Actinobacteriota | % | 72 | 0.14 | 0.44 | 0.26 | 0.06 | 0.23 |
|  | Proteobacteria | % | 72 | 0.13 | 0.33 | 0.21 | 0.04 | 0.17 |
|  | Acidobacteriota | % | 72 | 0.09 | 0.28 | 0.20 | 0.04 | 0.19 |
|  | Chloroflexi | % | 72 | 0.05 | 0.10 | 0.07 | 0.01 | 0.15 |
|  | Planctomycetota | % | 72 | 0.03 | 0.09 | 0.06 | 0.01 | 0.23 |
|  | Chloroflexi | % | 72 | 0.01 | 0.18 | 0.04 | 0.03 | 0.64 |
|  | Myxococcota | % | 72 | 0.01 | 0.05 | 0.03 | 0.01 | 0.35 |
|  | Gemmatimonadota | % | 72 | 0.02 | 0.04 | 0.03 | 0.00 | 0.17 |
|  | Bacteroidota | % | 72 | 0.01 | 0.06 | 0.02 | 0.01 | 0.39 |
|  | Methylomirabilota | % | 72 | 0.01 | 0.04 | 0.02 | 0.01 | 0.45 |
|  | others | % | 72 | 0.03 | 0.11 | 0.07 | 0.02 | 0.24 |
| Soil Fungi | Ascomycota | % | 72 | 0.50 | 0.99 | 0.75 | 0.12 | 0.16 |
|  | Basidiomycota | % | 72 | 0.01 | 0.32 | 0.08 | 0.06 | 0.73 |
|  | Mortierellomycota | % | 72 | 0.00 | 0.18 | 0.05 | 0.04 | 0.77 |
|  | unclassified_k__Fungi | % | 72 | 0.00 | 0.17 | 0.04 | 0.03 | 0.77 |
|  | Rozellomycota | % | 72 | 0.00 | 0.28 | 0.03 | 0.06 | 1.78 |
|  | Glomeromycota | % | 72 | 0.00 | 0.23 | 0.02 | 0.03 | 1.43 |
|  | Chytridiomycota | % | 72 | 0.00 | 0.09 | 0.01 | 0.01 | 1.65 |
|  | Calcarisporiellomycota | % | 72 | 0.00 | 0.06 | 0.00 | 0.01 | 3.11 |
|  | Olpidiomycota | % | 72 | 0.00 | 0.05 | 0.00 | 0.01 | 3.39 |
|  | Kickxellomycota | % | 72 | 0.00 | 0.01 | 0.00 | 0.00 | 1.33 |
|  | others | % | 72 | 0.00 | 0.04 | 0.00 | 0.00 | 3.81 |
| Soil Bacteria | Chao index | - | 72 | 30.00 | 44.00 | 34.90 | 2.93 | 0.08 |
|  | Shannon index | - | 72 | 1.78 | 2.27 | 2.14 | 0.10 | 0.04 |
|  | Simpson index | - | 72 | 0.14 | 0.25 | 0.17 | 0.02 | 0.13 |
|  | Pielou_e index | - | 72 | 0.52 | 0.65 | 0.61 | 0.03 | 0.04 |
| Soil Fungi | Chao index | - | 72 | 8.00 | 13.00 | 9.60 | 1.08 | 0.11 |
|  | Shannon index | - | 72 | 0.08 | 1.44 | 0.86 | 0.32 | 0.38 |
|  | Simpson index | - | 72 | 0.32 | 0.98 | 0.61 | 0.16 | 0.27 |
|  | Pielou_e index | - | 72 | 0.04 | 0.63 | 0.38 | 0.14 | 0.37 |

Note: The abbreviated meanings of soil properties are shown in Table S1. RB refers to the root biomass (kg m^-3^); RD refers to root average diameter (mm); RLD refers to the root length density (km m^-3^); RAD refers to the root surface area density (m^2^ m^-3^); SRL refers to specific root length (m g^-1^); SRA refers to specific root surface area (cm^2^ mg^-1^); RTD refers to root tissue density (g cm^-3^); RCC refers to root carbon content (%), and RNC refers to root nitrogen content (%).

**Table S3** Correlation analysis of soil properties, root traits and forest age

| Variable | Soil properties | | Variable | Root traits | |
| --- | --- | --- | --- | --- | --- |
|  | R^2^ | *P* |  | R^2^ | *P* |
| WC | 0.281 | **0.017** | RB | 0.207 | 0.081 |
| BD | -0.170 | 0.154 | RD | 0.327 | **0.005** |
| SP | 0.002 | 0.984 | RAD | 0.186 | 0.120 |
| pH | 0.088 | 0.460 | SRL | -0.158 | 0.186 |
| EC | -0.169 | 0.155 | SRA | -0.085 | 0.480 |
| TN | 0.518 | **0.000** | RTD | -0.056 | 0.642 |
| TOC | 0.565 | **0.000** | RNC | -0.152 | 0.203 |
| TP | -0.389 | **0.001** | RCC | -0.416 | **0.000** |
| C/N | 0.263 | **0.026** |  |  |  |
| C/P | 0.632 | **0.000** |  |  |  |
| N/P | 0.587 | **0.000** |  |  |  |
| Clay | 0.338 | **0.004** |  |  |  |
| Silt | 0.309 | **0.008** |  |  |  |
| Sand | -0.319 | **0.006** |  |  |  |

Note: The abbreviated meanings are shown in Table S1 and Table S2.

**Table S4** Multivariate ANOVA for the effects of species, forest age, soil depth, and interactions on microbial communities and diversity indies. The table displays the significance P-value.

| Variable | Species | Age | Depth | Species×  Age | Species×  Depth | Age×  Depth | Species×  Age×Depth |
| --- | --- | --- | --- | --- | --- | --- | --- |
| **Bacteria** |  |  |  |  |  |  |  |
| Actinobacteriota | **< 0.001** | 0.068 | **< 0.001** | **< 0.001** | **0.003** | 0.884 | 0.207 |
| Proteobacteria | **0.004** | **< 0.001** | **< 0.001** | **< 0.001** | **0.02** | 0.152 | 0.114 |
| Acidobacteriota | **< 0.001** | **< 0.001** | **< 0.001** | **< 0.001** | 0.482 | 0.183 | 0.296 |
| Chloroflexi | **< 0.001** | 0.081 | 0.835 | **< 0.001** | 0.254 | **0.022** | **0.021** |
| Planctomycetota | **< 0.001** | **< 0.001** | 0.115 | **< 0.001** | **0.042** | 0.752 | 0.221 |
| Chloroflexi | **< 0.001** | 0.777 | **0.014** | **< 0.001** | 0.138 | 0.154 | 0.105 |
| Myxococcota | 0.099 | **< 0.001** | **< 0.001** | **< 0.001** | 0.132 | 0.908 | 0.270 |
| Gemmatimonadota | **< 0.001** | **0.026** | **0.002** | **< 0.001** | **0.029** | 0.452 | **0.013** |
| Bacteroidota | **0.008** | **0.017** | **< 0.001** | **0.006** | **0.016** | **0.002** | 0.773 |
| Methylomirabilota | **< 0.001** | **< 0.001** | **< 0.001** | **< 0.001** | 0.063 | **< 0.001** | **0.016** |
| others | **0.033** | **0.003** | 0.955 | **< 0.001** | **0.025** | 0.290 | 0.099 |
| **Fungi** |  |  |  |  |  |  |  |
| Ascomycota | **< 0.001** | **< 0.001** | 0.195 | **0.019** | 0.576 | 0.918 | 0.758 |
| Basidiomycota | **0.016** | **0.003** | 0.395 | **0.016** | 0.562 | 0.946 | 0.527 |
| Mortierellomycota | **< 0.001** | 0.212 | **0.027** | **< 0.001** | **0.001** | **0.004** | 0.250 |
| unclassified_k__Fungi | **< 0.001** | **0.007** | 0.548 | **0.001** | 0.443 | 0.102 | 0.386 |
| Rozellomycota | **0.024** | **< 0.001** | **0.011** | **0.014** | 0.693 | **0.007** | 0.962 |
| Glomeromycota | **< 0.001** | **0.004** | 0.159 | **0.027** | 0.131 | 0.225 | 0.165 |
| Chytridiomycota | 0.119 | 0.515 | 0.763 | 0.088 | 0.440 | 0.677 | 0.814 |
| Calcarisporiellomycota | **0.008** | **0.001** | 0.545 | **0.002** | 0.872 | 0.902 | 0.989 |
| Olpidiomycota | **0.002** | **0.005** | 0.388 | **< 0.001** | 0.230 | 0.329 | 0.520 |
| Kickxellomycota | 0.259 | **0.008** | **< 0.001** | 0.288 | 0.305 | 0.105 | 0.132 |
| others | 0.179 | 0.354 | 0.229 | 0.107 | 0.317 | 0.304 | 0.243 |
| **Bacteria** |  |  |  |  |  |  |  |
| Chao index | 0.215 | 0.611 | **0.025** | **0.009** | **0.025** | 0.354 | 0.545 |
| Shannon index | **< 0.001** | **< 0.001** | 0.659 | **< 0.001** | **0.001** | 0.409 | 0.097 |
| Simpson index | **< 0.001** | **< 0.001** | 0.178 | **< 0.001** | **0.015** | 0.505 | 0.054 |
| Pielou_e index | **< 0.001** | **< 0.001** | 0.620 | **< 0.001** | 0.058 | 0.638 | 0.053 |
| **Fungi** |  |  |  |  |  |  |  |
| Chao index | 0.546 | **< 0.001** | 0.722 | 0.839 | 0.578 | 0.578 | 0.758 |
| Shannon index | **< 0.001** | **< 0.001** | 0.241 | **0.007** | 0.634 | 0.505 | 0.641 |
| Simpson index | **< 0.001** | **< 0.001** | 0.279 | **0.006** | 0.589 | 0.714 | 0.731 |
| Pielou_e index | **< 0.001** | **< 0.001** | 0.248 | **0.003** | 0.608 | 0.451 | 0.608 |

**
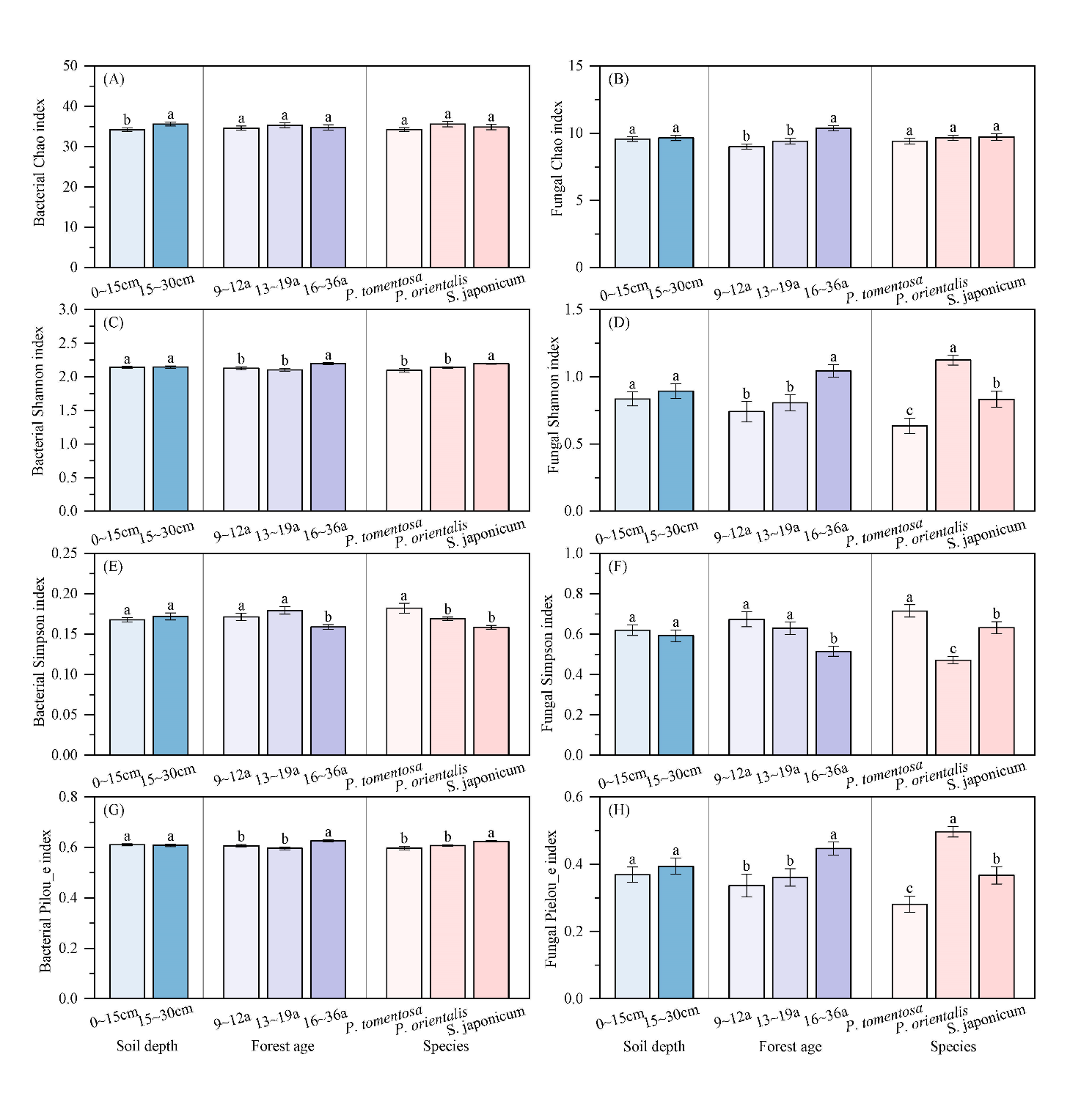
**

**Figure S1** The main effect of soil depth, forest age, and species on the Alpha diversity indies of bacterial (A, C, E, and G) and fungal (B, D, F, and H) communities on phylum level. Bars and errors show means ± SE. Bars sharing the same letter are not different between treatments at *P* < 0.05.

**
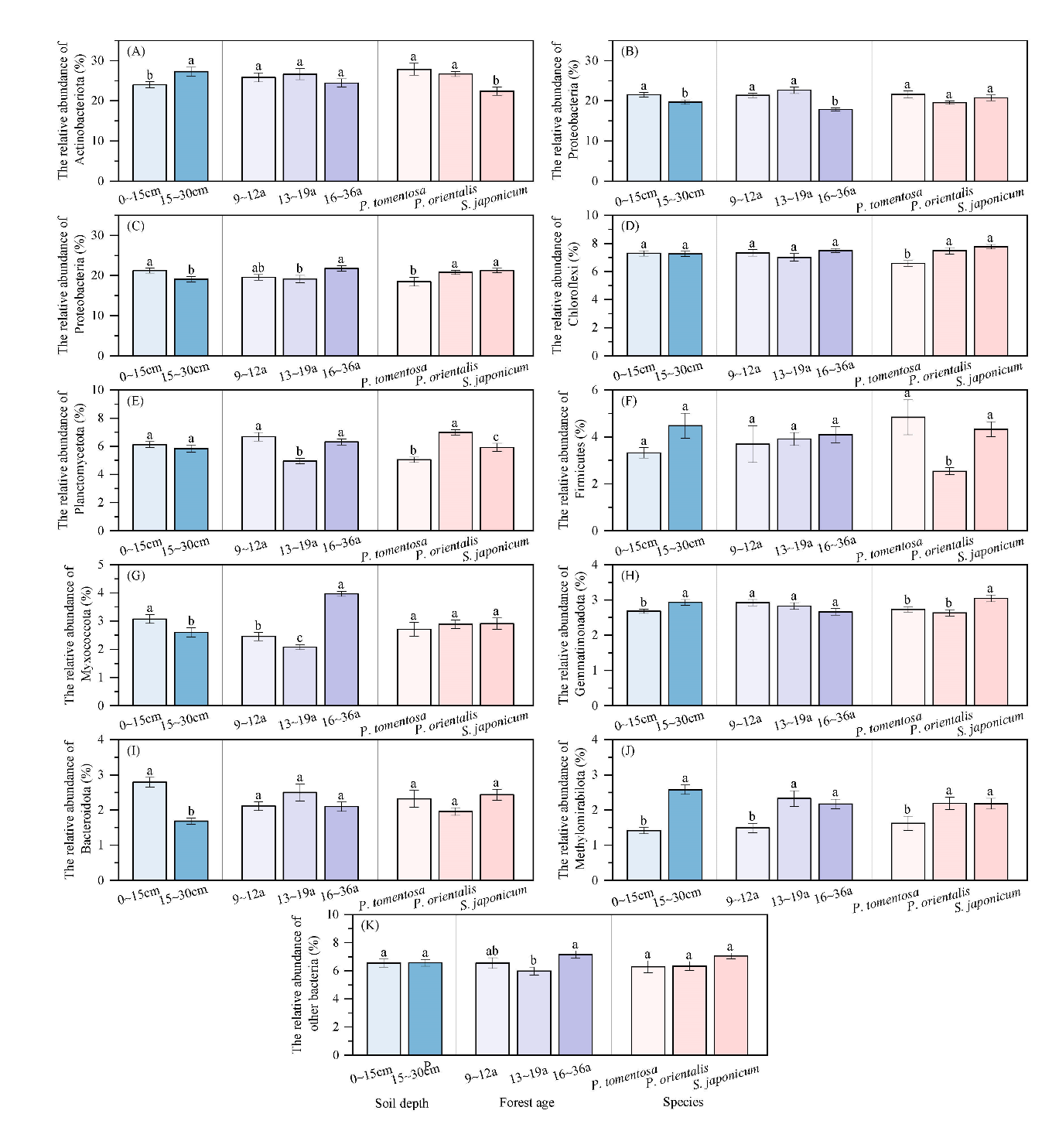
**

**Figure S2** The main effect of soil depth, forest age, and species on the relative abundance of each bacterial species on phylum level. Bars and errors show means ± SE. Bars sharing the same letter are not different between treatments at *P* < 0.05.


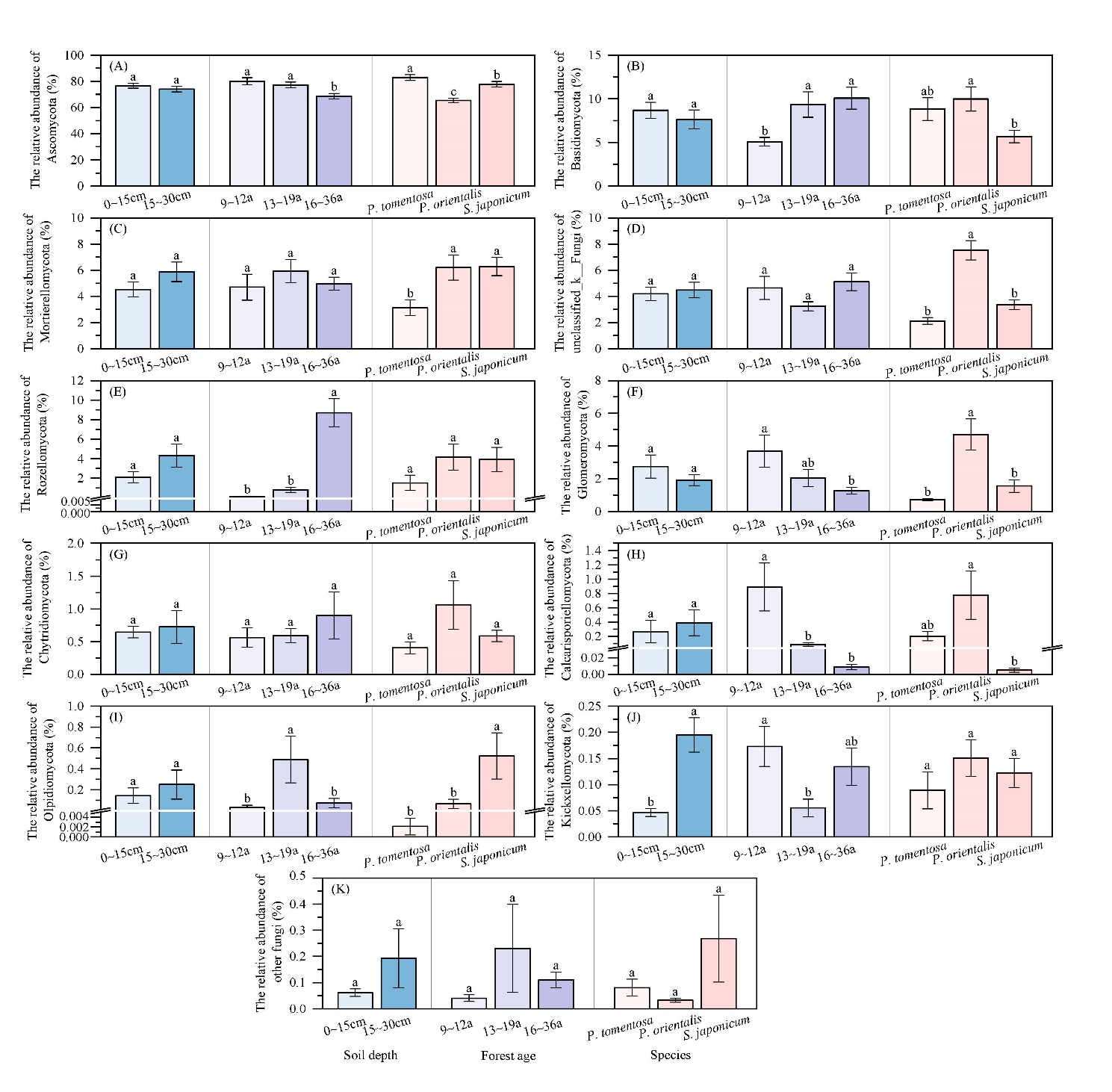


**Figure S3** The main effect of soil depth, forest age, and species on the relative abundance of each fungal species on phylum level. Bars and errors show means ± SE. Bars sharing the same letter are not different between treatments at *P* < 0.05.


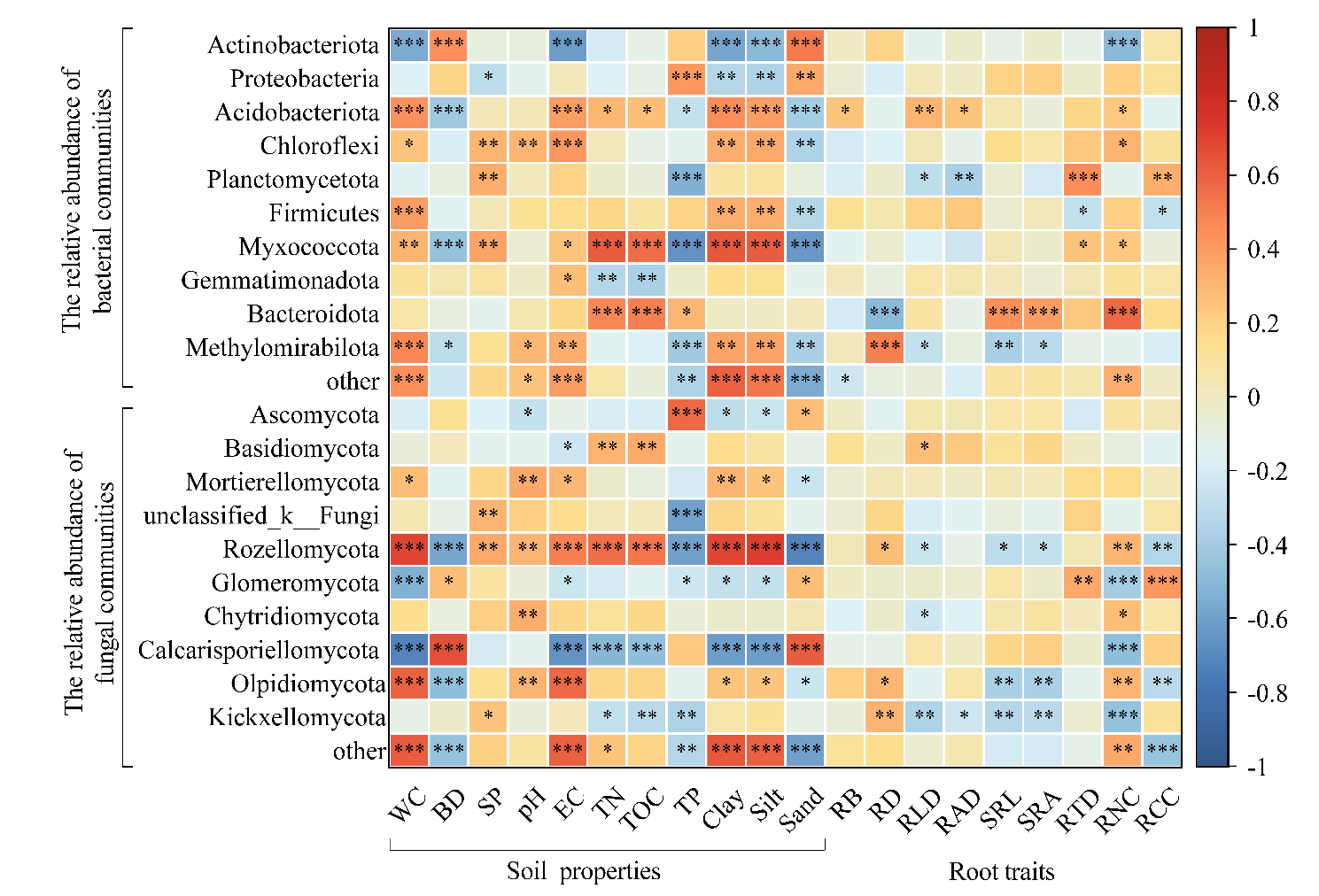


**Figure S4** Correlation analysis between the relative abundance of bacteria and fungi communities and factors (soil properties and root traits). The significant effects were reported at *P* < 0.05 (*), *P* < 0.01 (**) or *P* < 0.001(***) levels. The abbreviation explanations of soil properties and root traits are shown in the table 1.
